# The effect of interspecific competition on the temporal dynamics of *Aedes albopictus* and *Culex pipiens*

**DOI:** 10.1186/s13071-017-2041-8

**Published:** 2017-02-23

**Authors:** Giovanni Marini, Giorgio Guzzetta, Frederic Baldacchino, Daniele Arnoldi, Fabrizio Montarsi, Gioia Capelli, Annapaola Rizzoli, Stefano Merler, Roberto Rosà

**Affiliations:** 1 Department of Mathematics, University of Trento, Trento, Italy; 2 Department of Biodiversity and Molecular Ecology, Research and Innovation Centre, Fondazione Edmund Mach, San Michele all’Adige, Trento, Italy; 3 Fondazione Bruno Kessler, Trento, Italy; 4 Laboratory of Parasitology, Istituto Zooprofilattico Sperimentale delle Venezie, Padova, Italy

**Keywords:** Computational model, Mosquito population dynamics, Species interaction, Temporal shift, Vector abundance

## Abstract

**Background:**

*Aedes albopictus* and *Culex pipiens* larvae reared in the same breeding site compete for resources, with an asymmetrical outcome that disadvantages only the latter species. The impact of these interactions on the overall ecology of these two species has not yet been assessed in the natural environment. In the present study, the temporal patterns of adult female mosquitoes from both species were analysed in north-eastern Italy, and substantial temporal shifts between abundance curves of *Cx. pipiens* and *Ae. albopictus* were observed in several sites. To understand which factors can drive the observed temporal shifts, we developed a mechanistic model that takes explicitly into account the effect of temperature on the development and survival of all mosquito stages. We also included into the model the effect of asymmetric interspecific competition, by adding a mortality term for *Cx. pipiens* larvae proportional to the larval abundance of *Ae. albopictus* within the same breeding site. Model calibration was performed through a Markov Chain Monte Carlo approach using weekly capture data collected in our study sites during 2014 and 2015.

**Results:**

In almost half of observation sites, temporal shifts were due to competition, with an early decline of *Cx. pipiens* caused by the concurrent rise in abundance of its competitor, and this effect was enhanced by higher abundance of both species. We estimate that competition may reduce *Cx. pipiens* abundance in some sites by up to about 70%. However, in some cases temporal shifts can also be explained in the absence of competition between species resulting from a “temporal niche” effect, when the optimal fitness to environmental conditions for the two species are reached at different times of the year.

**Conclusions:**

Our findings demonstrate the importance of considering ecological interactions and, in particular, competition between mosquito species in temperate climates, with important implications for risk assessment of mosquito transmitted pathogens, as well as the implementation of effective control measures.

**Electronic supplementary material:**

The online version of this article (doi:10.1186/s13071-017-2041-8) contains supplementary material, which is available to authorized users.

## Background

A fundamental concept in ecology is that competition for limited resources can take place between individuals of the same species, i.e. intraspecific competition, or between individuals of different species, i.e. interspecific competition. In interspecific competition, individuals of different species compete for the same resource (e.g. food or living space) therefore limiting resource availability for the other species. Such competition could lead to the exclusion of the weaker species but also to coexistence equilibrium *via* different mechanisms, such as differential resource use and spatial or temporal variations in habitat conditions [1, 2].

Among mosquito species, interspecific competition plays a key role in structuring the community at the larval stage in water-filled containers [3]. In Europe, *Aedes albopictus* and *Culex pipiens* are two of the most widely spread mosquito species [4–6]. While *Cx. pipiens* is indigenous, *Ae. albopictus* is native to Asia and was introduced in several European countries at the end of the last century [5]; since then, *Ae. albopictus* rapidly spread in urban and suburban environments, occupying a habitat already exploited by *Cx. pipiens*. These two species are vectors of many arboviruses. *Ae. albopictus* can transmit dengue, chikungunya, Zika and West Nile viruses [7–12], while *Cx. pipiens* is the most important vector of West Nile virus in Europe [13]. Local vector abundance, which drives the pathogen-transmission dynamics [14, 15], might be influenced by interspecific competition. Furthermore, larval competition might have indirect effects on epidemiological risks by altering mosquito-virus interactions in adult females [16]; different *Aedes* mosquitoes (including *Ae. albopictus*, *Ae. aegypti* and *Ae. triseriatus*) bred in conditions of nutritional stress imposed by the interspecific competition were more susceptible to infection and more able to transmit various pathogens such as dengue, Sindbis and LaCrosse viruses [17–19]. In addition, adult survival, and consequently the length of infectious period for infected females (i.e. their vectorial capacity), might be influenced by interspecific interactions occurred at the larval stage [20, 21]. Therefore, competition may have important consequences on the epidemiology of mosquito-borne infections and their potential control strategies [3].

Previous laboratory studies show that *Ae. albopictus* is a strong competitor against other species; *Ae. albopictus* larvae have been shown to negatively affect the growth and survival of larvae from other mosquito species bred in the same site, including *Ae. aegypti* [22, 23], *Ae. japonicus* [24], *Ae. triseriatus* [25, 26], *Ae. koreicus* (F. Baldacchino, unpublished observations) and *Culex pipiens* [27, 28]. *Aedes albopictus* larvae, on the other hand, were substantially unaffected by the presence of *Cx. pipiens* larvae. This asymmetric interspecific competition has been attributed to a higher efficiency of *Ae. albopictus* in converting food to biomass [27]. The strength of competition effects has been shown to depend on food resource types [27] and temperature, with a maximal effect on *Cx. pipiens* larval mortality observed at temperatures above 25 °C [27].

In nature, *Ae. albopictus* and *Cx. pipiens* can exploit common water-filled containers as larval habitats. Generally, *Ae. albopictus* prefers ovipositing in small natural and artificial containers, while *Cx. pipiens* prefers larger water bodies [27, 29]. However, these two species can share medium size containers. In northern Italy, during entomological surveys in the summer of 1996 and 1997, *Ae. albopictus* and *Cx. pipiens* were detected together in 67% of larval habitats, especially drums, buckets, catch basins and tires [27]. Thus, coexistence between these two species could be shaped by both interspecific competition and niche differentiation involving temporal and spatial factors [2, 3].

The temporal patterns of local populations of *Ae. albopictus* and *Cx. pipiens* in northern Italy can be highly variable depending on climate and landscape [30, 31], but generally *Cx. pipiens* is active earlier than *Ae. albopictus* [27, 32]. *Culex pipiens* larvae appear in springtime and peak in July, while *Ae. albopictus* larvae appear several weeks after *Cx. pipiens* and peak in September [27]. Different temporal profiles may be driven by different life history strategies and patterns of survival, oviposition and egg hatching under variable environments [27, 28]. In the case of *Cx. pipiens*, only adult females undergo diapause, and shortening photoperiods induce diapause in a growing number of newly emerged adult females [33, 34]. Early in the mosquito breeding season, overwintering females of *Cx. pipiens* begin to lay eggs on water surface. In contrast, *Ae. albopictus* overwinters as diapausing eggs [29], which hatch several weeks after *Cx. pipiens* eggs; then newly emerged *Ae. albopictus* females lay eggs above the water line, and hatching is induced by submergence after precipitations. This asynchrony in hatching between the two species allows *Cx. pipiens* larvae to develop in the absence of *Ae. albopictus* and provides to *Cx. pipiens* a refuge from competition early in the season [28]. Furthermore, high temperatures observed in summertime decrease *Cx. pipiens* adult survival [35, 36], while *Ae. albopictus* is better adapted to warmer conditions [37]. Therefore, environmental conditions can create a “temporal niche” effect, allowing a shift in optimal breeding seasons of the two species. Nonetheless, the “temporal niche” effect is not always sufficient to explain the observed temporal profiles of competing mosquito species [2].

Disentangling the ecological mechanisms that drive mosquito population dynamics might be difficult with a simple statistical analysis of the observed capture pattern. However, mechanistic models incorporate a range of biological processes that drive mosquito population dynamics. Therefore, they are more suitable to investigate the main determinants of dynamical patterns, such as the effect of “temporal niches” and interspecific competition. Several mechanistic models have been used to describe the population dynamics of single mosquito species, including *Ae. albopictus* [38–40] and *Cx. pipiens* [41–43]. In this study, we describe and interpret, in a robust mathematical framework, observed differences in temporal patterns of *Ae. albopictus* and *Cx. pipiens*, aiming to disentangle the contribution of the “temporal niche” effect and interspecific competition on their population dynamics.

To this aim, we develop a mathematical model that describes temporal variations of population dynamics of both species and allows for interspecific interactions at the larval stages, as previously evaluated in laboratory conditions. To the best of our knowledge, this is one of the first efforts to assess the impact of *Ae. albopictus* and *Cx. pipiens* ecological interactions in natural conditions.

## Methods

### Study area and mosquito data

The study was carried out in the provinces of Belluno (46°08′27″N, 12°12′56″E) and Trento (46°04′00″N, 11°07′00″E), Northern Italy. This mountainous area covers a large part of the Dolomites and the Southern Alps. The climate is temperate-oceanic with four main areas: sub-Mediterranean (close to Lake Garda with mild winters), subcontinental (the main valleys with more severe winters), continental (the alpine valleys) and alpine (the areas above the tree line) [44].

**Fig. 1:**
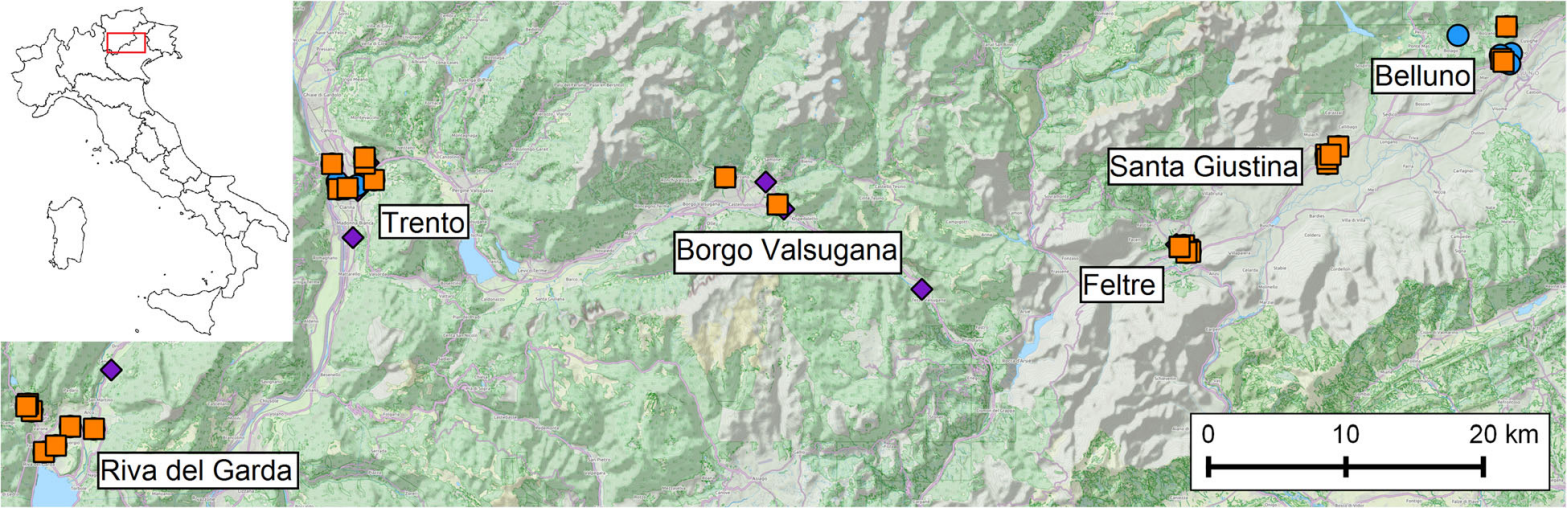
Map of the study area. Selected trap locations (*purple* diamonds: 2014; *blue* circles: 2015; *orange* squares: both years)

We performed entomological surveillance of several mosquito species in the provinces of Trento and Belluno during 2014 and 2015. Mosquitoes were collected using Biogents Sentinel traps (BG trap; Biogents AG, Regensburg, Germany) baited with commercial lures from the same producer and CO_2_ from dry ice. The BG trap has been demonstrated to collect a great variety of species and is efficient for both *Ae. albopictus* and *Cx. pipiens* when baited with CO_2_, especially in an urban environment [45]. Trapping locations were chosen within three municipalities of Belluno province (Belluno, Feltre, Santa Giustina) and three of Trento province (Borgo Valsugana, Riva del Garda, Trento) (see Fig. 1).

A total of 73 BG traps were located within urban and peri-urban areas at altitudes ranging from 75 m to about 640 m above sea level. As recommended by the manufacturer, traps were placed in shaded positions sheltered from wind and rainfall. In 2014, 39 traps were positioned while in 2015 the number of traps was reduced to 34; 28 trapping sites where shared among the two years. BG traps ran for 24 h, approximately every two weeks, from the end of April to the beginning of November. Mosquito sex and species were identified using taxonomic keys [46]. The number of capture sessions differed from trap to trap because of logistic reasons, ranging from a minimum of 8 to a maximum of 15.

### Delay analysis

We analysed temporal shifts between pairs of time series of captured female adults of *Cx. pipiens* and *Ae. albopictus,* observed at the same site and year. To this aim, we estimate the time lag *T* between the two time series at which the cross-correlation function *ρ* reaches its maximum (see [47] for details):

$$ T={\mathrm{argmax}}_{\tau}{\rho}_{XY}\left(\tau \right)=\underset{\tau}{\mathrm{argmax}}{\displaystyle {\sum}_{t=1}^m X\left[ t\right] Y\left[ t+\tau \right]} $$

More specifically, *ρ*_*XY*_[*τ*] is the cross-correlation function, *X* and *Y* are the time series for *Cx. pipiens* and *Ae. albopictus* respectively, *m* is the length of the time series expressed in weeks. *T* measures the time lag (in weeks) between the two time series and it is labelled throughout the manuscript as the “interspecific delay” between *Ae. albopictus* and *Cx. pipiens* temporal dynamics.

### Environmental data

Original land surface temperature (LST) data were obtained from the MODIS version 5 LST products MOD11A1 and MYD11A1 [48]. We used the average daily temperature and a spatial resolution of 250 m [49].

**Fig. 2:**
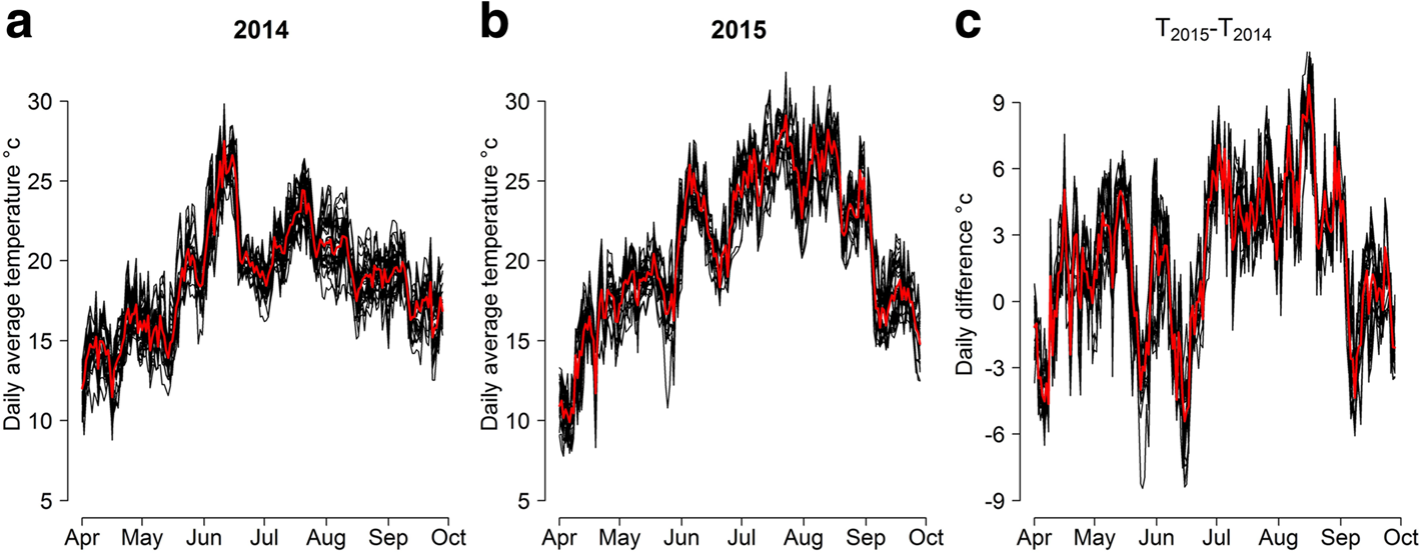
Daily temperature at study sites. **a** Daily average temperatures from the 39 trap locations in 2014 (*black lines*) and average across all sites (*red line*); **b** daily average temperatures from the 34 trap locations in 2015 (*black lines*) and average across all sites (*red line*); **c** daily average temperature difference between 2015 and 2014 from the 28 sites represented in both years

There was a striking difference in recorded temperatures between the two considered years (Fig. 2a, b); specifically, for the 28 sites sampled in both years, the difference in the average daily temperature during summer months (July to September) between 2014 and 2015 (Fig. 2c) is close to 5 °C, with average observed temperatures of 20.6 °C and 25.5 °C for the two years, respectively. For comparison, the average temperature for this period computed over years 2004–2013 is about 21.9 °C [50], so that the two years of study represent the extremes of a wide range of possible temperature scenarios. Daylight lengths for the centroid of the study region during the considered period were obtained from the US Naval Observatory [51].

### Population model

We developed a mathematical model of the abundance of the two vector species based on the *Ae. albopictus* model proposed in [39, 52, 53] and on the *Cx. pipiens* model proposed in [43]. The original models account for the population dynamics of each species by considering temperature-dependent development and mortality rates and intraspecific larval density dependent factors; the model for *Cx. pipiens* also includes a photoperiod-dependent diapause rate for pupae. Here, we include the effect of asymmetric interspecific competition, by adding a mortality term for *Cx. pipiens* larvae proportional to the larval abundance of *Ae. albopictus* in the same site. Given that the effect of interspecific competition weakens for temperatures below 20 °C [27], we set the competition coefficient to zero for average daily temperatures less than 15 °C, and to a constant value, estimated *via* model calibration, otherwise.

The model was calibrated using a Markov Chain Monte Carlo (MCMC) approach applied to the Poisson likelihood of observing the empirical capture data, given the model-predicted abundance. The model has five free model parameters: two daily capture rates of adult mosquitoes (one for *Ae. albopictus* and one for *Cx. pipiens*); an intraspecific larval density dependent factor for each species, representing the availability of suitable breeding sites and food resources at a given site; and the interspecific larval competition coefficient, which increases the mortality of *Cx. pipiens* larvae proportionally to the abundance of *Ae. albopictus* larvae. The posterior distribution of parameters was obtained by a random-walk Metropolis-Hastings sampling [54], using uniform prior distributions and normal jumps.

To evaluate the hypothesis of interspecific competition, we calibrated a simplified model representing the assumption of independent populations, where the larval competition coefficient was fixed to zero. We then compared the goodness of fit of the two models (with and without competition) using the Deviance Information Criterion (DIC) [55, 56]. The model including competition was preferred to the model with independent populations when its DIC value was lower by a minimum threshold, which was conservatively set to four (compared to a minimum recommended threshold of 2 [55]).

All relevant details on model equations, calibration procedure, model selection and sensitivity analysis of results with respect to model selection criteria are reported in the Additional file 1: Database 1 (see sections “Model calibration” and “DIC and AIC analysis”).

## Results

**Fig. 3:**
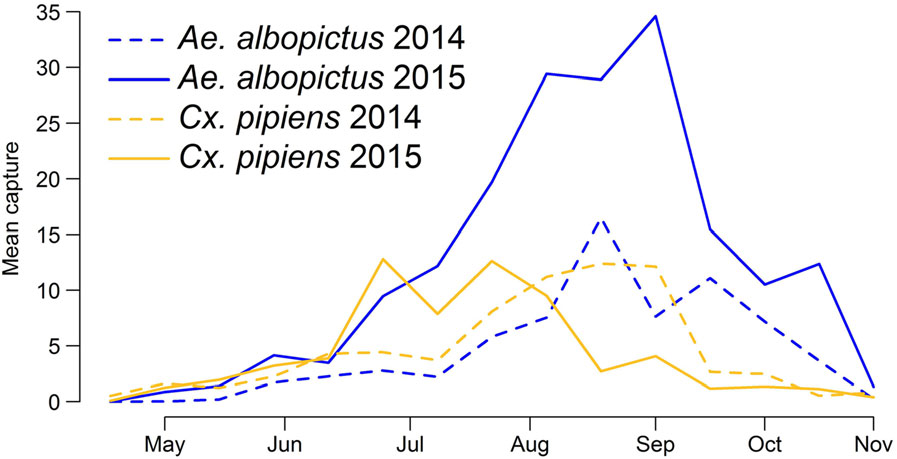
Mosquito temporal dynamics. Number of adult female mosquito captures for the two species (*Ae. albopictus* in *blue* and *Cx. pipiens* in *yellow*), averaged over all sites, for 2014 and 2015 (dashed and continuous lines respectively)

The total number of trapped female *Ae. albopictus* and *Cx. pipiens* in all sites and years was 4,566 and 8,362, respectively. Capture data are presented in the Additional file 2: Database S2. As shown in Fig. 3, *Cx. pipiens* abundance was similar between the two years (54% of total captured in 2014) while the majority of *Ae. albopictus* were captured in 2015 (69% of total). This is likely because 2015 was warmer and therefore more suitable for *Ae. albopictus*.

*Culex pipiens* shows different patterns between the two considered years. In fact, in 2015 *Cx. pipiens* abundance starts declining earlier in the season, in conjunction with the increase of *Ae. albopictus* abundance, while in 2014 the two species show a more synchronous pattern (Fig. 3). On the other hand, temporal dynamics of *Ae. albopictus* do not show substantial inter-annual differences (Fig. 3).

**Fig. 4:**
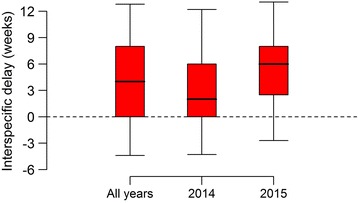
Interspecific delay. The interspecific delay (in weeks, median, quartiles and 95% quantiles) computed for *Ae. albopictus* and *Cx. pipiens* capture patterns. Distributions are shown for all time series combined and aggregated by year

**Fig. 5:**
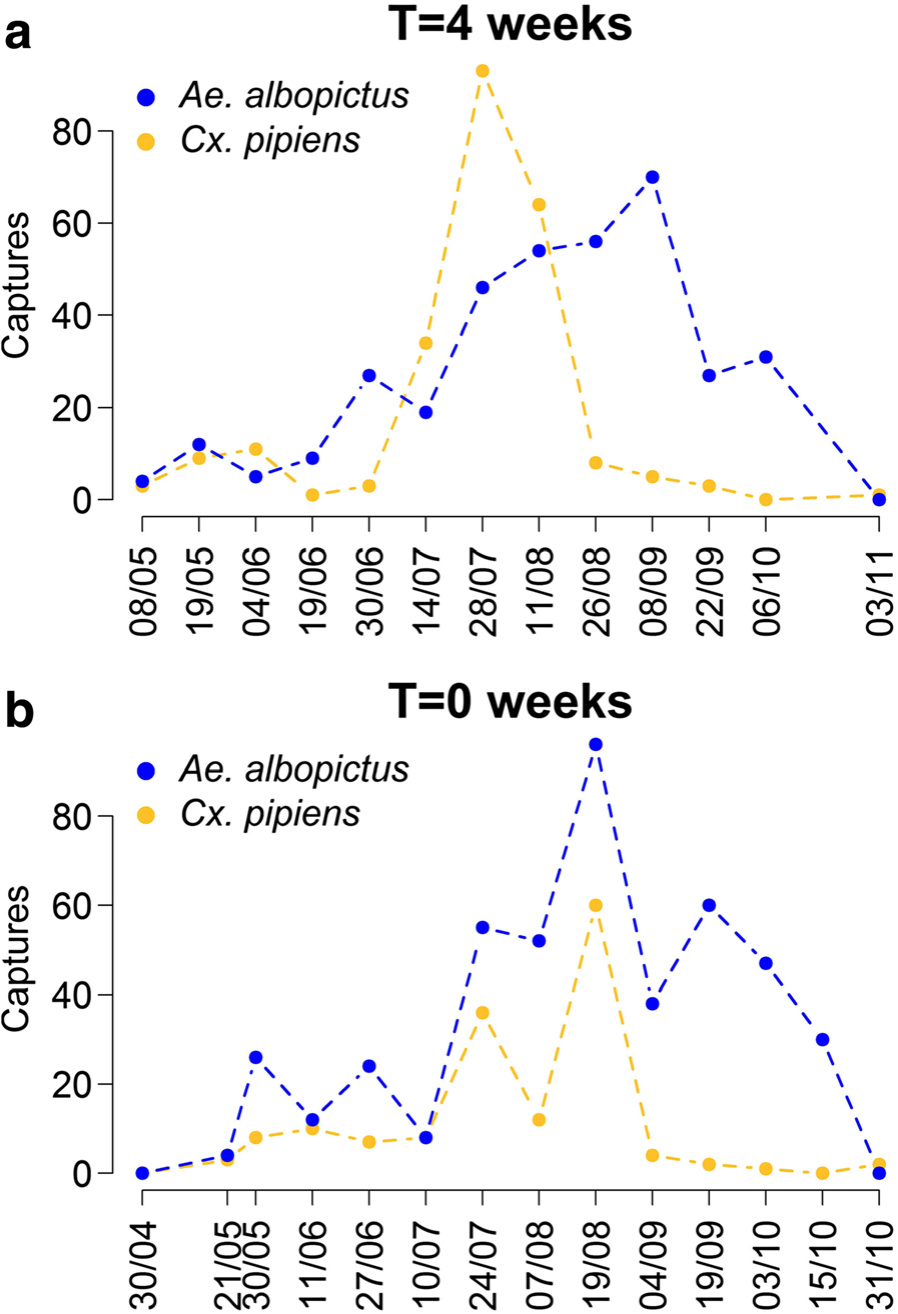
Two examples of recorded temporal patterns with different interspecific delay. T = 4 weeks (**a**), no delay, i.e. T = 0 (**b**). *Aedes albopictus* (*blue*) and *Cx. pipiens* (*yellow*) recorded captures from two datasets

Figure 4 presents the distribution of interspecific delays computed over all available time series. We can note that temporal profiles of *Ae. albopictus* are delayed, with respect to *Cx. pipiens* from the same site and year, by more than 4 weeks (29 days) on average; a higher delay was recorded in 2015 (37 days) with respect to 2014 (22 days) (*t*-test *t* = -1.9912, *df* = 69.597, *P* = 0.050) (Fig. 4). Figure 5 illustrates two examples of observed capture patterns associated with an average delay of four weeks (Fig. 5a) and no delay (Fig. 5b).

For 29 time series (around 40%) the model with competition assumption was better (ΔDIC > 4) at explaining the observed capture dynamics; of these, the large majority (22) were time series from 2015 (see Table 1). On the other hand, 44 time series (around 60%) were better described (ΔDIC < 4) by the model without the competition assumption; of these, 32 were time series observed in 2014 (Table 1). An overall comparison of selected model fits for all time series can be seen in the Additional file 3: Figure S1.

**Table 1:** Number of time series by year and selected model. Time series, classified according to model selection based on the Deviance Information Criterion, are shown for grouped and separated years. Percentages are computed by row

	All time series	Competition (%)	Independent populations (%)
All years	73	29 (40)	44 (60)
2014	39	7 (18)	32 (82)
2015	34	22 (65)	12 (35)

**Fig. 6:**
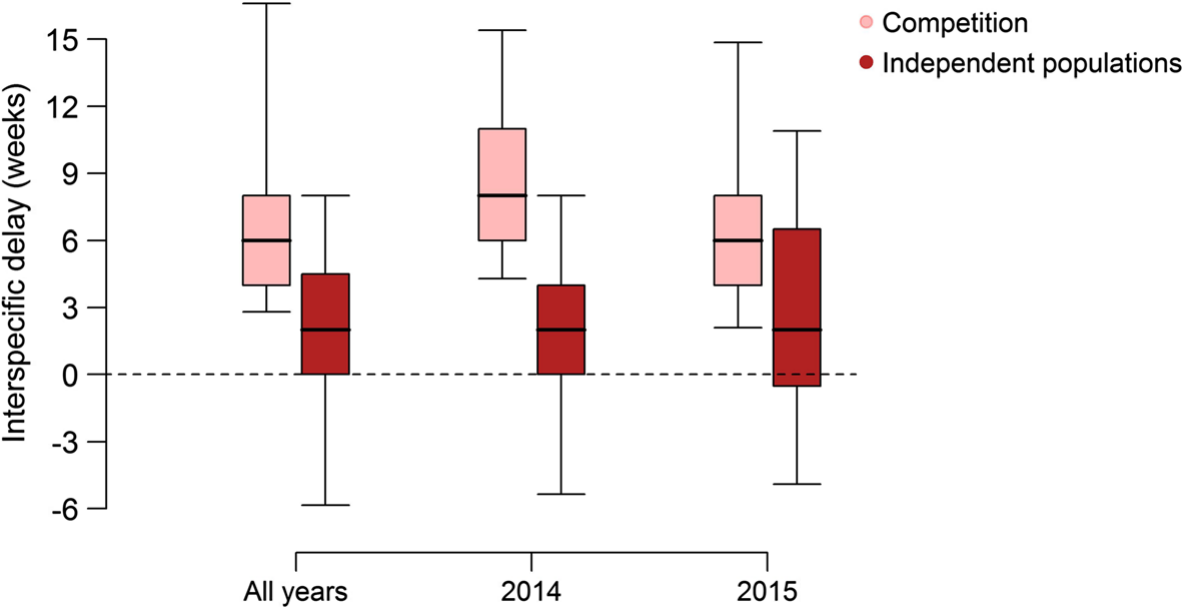
Interspecific delay by selected model. The interspecific delay (in weeks, median, quartiles and 95% quantiles) computed for *Ae. albopictus* and *Cx. pipiens* capture patterns by the selected model. Distributions are shown for all time series combined and aggregated by year

In Fig. 6, we show the distribution of interspecific delays aggregated by the selected model (competition *vs* independent populations) and by year. In sites with competition, *Ae. albopictus* capture patterns had a systematic and large positive delay with respect to *Cx. pipiens* (average 51 days considering the two years combined). Conversely, in sites where the independent population model prevailed, the average interspecific delay was lower (14 days considering the two years combined). There were no significant differences between average delays from the same group and different years.

**Fig. 7:**
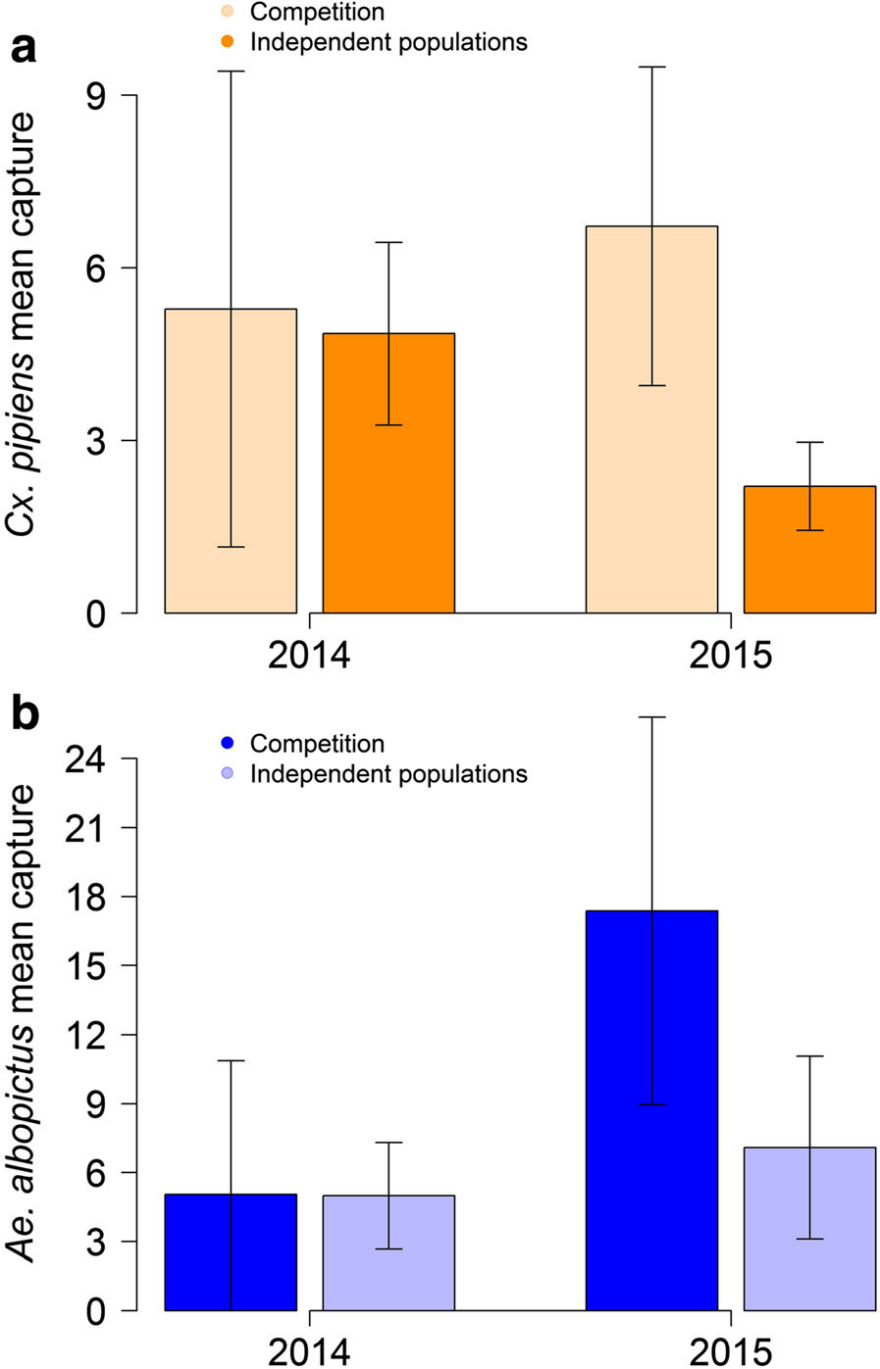
Average recorded captures by selected model. Average number of captured *Cx. pipiens* (**a**) and *Ae. albopictus* (**b**) per site by selected model and year. Black lines represent the 95% confidence intervals

We analysed the average numbers of mosquitoes captured per session and site, aggregated by selected model and year (Fig. 7). In 2014, when competition was rare, abundances of *Cx. pipiens* and *Ae. albopictus* were not significantly different within competition time series compared to those without; however, in 2015 competition was much more common, and both *Ae. albopictus* and *Cx. pipiens* were significantly more abundant within competition sites (*t*-test *t* = -3.2873, *df* = 23.758, *P* = 0.003). This result suggests that high mosquito densities might have increased the chance of competition in 2015, possibly because of the increased likelihood of shared oviposition sites. Interestingly, despite the higher mortality of *Cx. pipiens* larvae in competition sites, we did not find an overall reduction in *Cx. pipiens* captures in 2015 respect to 2014 within competition sites. This seemingly counterintuitive result can be better interpreted by considering temporal dynamics (Fig. 3): in the early part of the 2015 season, *Cx. pipiens* were much more abundant than in the same period of 2014, because of improved environmental conditions; however, with the rapid expansion of *Ae. albopictus*, the 2015 abundance of *Cx. pipiens* fell briskly, offsetting the advantage of the first part of the season. Considering only sites with competition, the model predicted a reduction for the average *Cx. pipiens* abundance by 49.2% (95% quantiles: 14–74%), compared to corresponding simulations obtained with independent populations. The onset of competition effects (defined as the first date at which relative differences between the *Cx. pipiens* populations predicted by models with and without competition exceed 10%) ranged from the end of April to the middle of July, with median centred on the first ten days of June.

## Discussion

In this study, we analysed time series of *Ae. albopictus* and *Cx. pipiens* captures in northern Italy. The observed dynamics of the two species show, in several cases, a marked misalignment in temporal patterns, with a delay of *Ae. albopictus* abundance patterns with respect to *Cx. pipiens*. We showed that these temporal shifts could be explained by two alternative mechanisms: temporal niche effects and asymmetric interspecific larval competition. Under the assumption that the two populations do not interfere with each other in a given area, a temporal niche effect may occur, depending on environmental variables (e.g. temperature, photoperiod), when the two species reach their peak of abundance at different times of the year. On the other hand, in general, competition causes more pronounced delays between the dynamics of the two species. That is due to an increase of *Cx. pipiens* larval mortality in the presence of *Ae. albopictus* within the same breeding site, inducing an anticipated decline of *Cx. pipiens* adult population.

An asymmetric competition between *Ae. albopictus* and *Cx. pipiens* has been observed and replicated in several laboratory experiments [27, 28], but its importance in the natural environment has not been assessed before. According to our modelling results, asymmetric interspecific competition explained the seasonal patterns of *Cx. pipiens* well in many sampling sites. Specifically, in 2015 the model with competition assumption better described empirical observations in 65% of the considered sites, compared to only 18% in 2014. Our results suggest that this difference was associated with higher temperatures in 2015 than in 2014, which caused both a direct increase in the competition effect [27] and an increase of *Ae. albopictus* densities due to a considerable reduction in the development time of immature stages [37]. High *Ae. albopictus* densities may increase the chance of oviposition in containers shared with *Cx. pipiens* [21]. In addition, drier conditions observed in summer 2015 (about 122 mm total precipitations on average, compared to 355 mm in 2014 [50]) likely reduced the number of small rain-filled containers available for *Ae. albopictus*, pushing females to oviposit in medium containers with the presence of *Cx. pipiens* larvae. Thus, the larval habitats of *Ae. albopictus* and *Cx. pipiens* might have overlapped more under such conditions, leading to a greater frequency of interspecific competition [27]. Abiotic factors in 2015 were also more favourable for *Cx. pipiens*, but the adverse effects of competition strongly limited their increase with respect to 2014. We estimated a relative reduction of *Cx. pipiens* abundance due to competition of about 50% on average (and up to 70% in some sites), compared to equivalent environmental conditions where competition was discounted.

Under natural conditions, competition occurs within breeding sites where the two species happen to oviposit together, mainly in urban and peri-urban areas. Adults of *Ae. albopictus* and *Cx. pipiens* captured in a given trap emerged from various breeding sites present in the neighbourhood of the trap location. Among these breeding sites, some are colonized by a single larval species, while others are colonized by both species, where they then compete for resources. Therefore, our sampling procedure can only weigh the average effect of competition in the neighbourhood of the trapping site. Our criterion for model selection was designed conservatively to identify sites where competition has a strong overall effect on the temporal patterns of mosquito abundance (see section “Model fit” in the Additional file 1: Database S1). It is likely that competition occurs, to a lower degree, even in trapping sites that were not classified as “competition” sites. To explore the robustness of results with respect to the model selection criterion, we did a sensitivity analysis by using different score functions for model selection and considering different threshold values. Results confirmed our main conclusions and suggested that competition may have shaped *Cx. pipiens* dynamics in 30 to 50% of all time series (see section “DIC and AIC analysis” in the Additional file 1: Database 1 for details).

For the sake of simplicity, in this study we assumed competition would affect only *Cx. pipiens* larval survival. It has been shown that interspecific competition at this stage might also increase *Cx. pipiens* larval developmental time [28] and reduce the body size of newly emerged adults [21, 27], with possible negative implications for female fecundity and longevity [21]. Moreover, the body size has been associated with female susceptibility to virus infection [16, 19], and female longevity is a main component of the vectorial capacity. Non-lethal effects are more likely to have an impact at low densities of *Ae. albopictus* larvae [27, 28], i.e. in situations where the independent population hypothesis could not be rejected by the present analysis, or at poor food resource conditions [21]. Thus, the inclusion of non-lethal effects in the competition model might unveil an even more widespread importance of competition effects on vector populations and arbovirus transmission. However, abundance data provided by mosquito trapping only give information on the cumulative effects of competition on the adult population; therefore, they are insufficient to distinguish the relative contribution of different competition effects. We chose to only model increased larval mortality, being the strongest competition outcome [27, 28] and the one most directly affecting the adult population. Nonetheless, quantitative experiments collecting further data on non-lethal competition effects might improve the development of models and foster our understanding of ecological mechanisms. In addition, given the importance of climatic factors in shaping mosquito population dynamics, important additional insights will be needed, from further experiments, to quantify the effect of temperature on the strength of lethal and non-lethal effects of interspecific competition [27].

## Conclusions

We found that interspecific competition between *Cx. pipiens* and *Ae. albopictus* is common in temperate climates and it is enhanced by higher mosquito densities produced by higher temperatures. Drier weather conditions may also induce a higher overlap of breeding sites for different mosquito species, increasing the overall chances for competition. We have shown that competition amplifies the temporal separation between seasonal patterns of the two species, with *Cx. pipiens* arising early and declining more quickly with the rise of *Ae. albopictus*. Finally, we have shown that competition induces significant reductions in the total abundance of *Cx. pipiens*.

Understanding the interaction between climatic variables, competition and resulting vector abundances can be important to improve our estimates of epidemiological risks for arboviruses for which *Cx. pipiens* and *Ae. albopictus* are competent vectors, and for the assessment of vector control strategies [57, 58]. Furthermore, recent findings have shown that interspecific competition at the larval stage may strongly affect the viral competence of adult mosquitoes [16] as well as their vectorial capacity by modifying their longevity [20, 21]. If similar effects exist in the competition between *Ae. albopictus* and *Cx. pipiens*, they would significantly impact the viral susceptibility and transmission potential of local mosquito populations and should therefore be considered in the estimation of outbreak risks [52, 53].

## Additional files

Additional file 1:
**Database S1.** Supporting text containing methodological details and additional results. (PDF 872 kb)
Additional file 2:
**Database S2.** Observed time series. Datasets used for model calibration: trapped adult mosquitoes of both species. (XLSX 18 kb)
Additional file 3:
**Figure S1.** Model fit for *Cx. pipiens* (*orange*) and *Ae. albopictus* (*blue*) in the 73 datasets. Left side: datasets from 2014; sites where the competition model was selected are shown in the first two rows. Right side: datasets from 2015; sites where the competition model was selected are shown in the first six rows. Squares: capture data; lines and shaded areas: posterior model estimates with 95% credible intervals. (JPG 19486 kb)
