# Supplementary material for: The effect of interspecific competition on the temporal dynamics of Aedes albopictus and Culex pipiens
Source: Parasit Vectors. 2017 Feb 23;10:102. doi: 10.1186/s13071-017-2041-8 (PMC5322594; doi:10.1186/s13071-017-2041-8)
Supplement: Additional file 1: — Database S1. Supporting text containing methodological details and additional results. (PDF 872 kb) [file 13071_2017_2041_MOESM1_ESM.pdf]

The effect of interspecific competition on the temporal dynamics of *Aedes albopictus* and *Culex pipiens*

Giovanni Marini [1, 2¶*], Giorgio Guzzetta [3¶], Frederic Baldacchino [2], Daniele Arnoldi [2], Fabrizio Montarsi [4], Gioia Capelli [4], Annapaola Rizzoli [2], Stefano Merler [3], Roberto Rosà[2]

1: Department of Mathematics, University of Trento, Italy;
2: Department of Biodiversity and Molecular Ecology, Research and Innovation Centre, Fondazione Edmund Mach, San Michele all'Adige (TN), Italy;
3: Fondazione Bruno Kessler, Trento, Italy;
4: Laboratory of Parasitology, Istituto Zooprofilattico Sperimentale delle Venezie, Padova, Italy;

¶These authors contributed equally to this work
*Corresponding author

Appendix

Model calibration

The model accounts for the seven *Cx. pipiens* and four *Ae. Albopictus,* life stages of, namely eggs (E_c and E_a), the four *Cx. pipiens* larval instars (L_{1c}, L_{2c}, L_{3c}, L_{4c}) and one *Ae. albopictus* larval stage (L_a), pupae (P_c and P_a), non-diapausing *Cx.* female adults (A_c) and *Ae.* female adults (A_a). The ODE version of the model is based on the following system of equations:

$$
\begin{aligned}
E'_c &= \frac{n_{E_c}}{d_{A_c}} A_c - \left(\mu_{E_c} + \tau_{E_c}\right) E_c \\
E'_a &= \frac{n_{E_a}}{d_{A_a}} A_a - \left(\mu_{E_a} + \tau_{E_a}\right) E_a \\
L'_{1_c} &= \tau_{E_c} E_c - \left(\tau_{L_{1_c}} + \mu_{L_{1_c}} \left(1 + \frac{L_c + \alpha L_a}{K_c}\right)\right) L_{1_c} \\
L'_{2_c} &= \tau_{L_{1_c}} L_{1_c} - \left(\tau_{L_{2_c}} + \mu_{L_{2_c}} \left(1 + \frac{L_c + \alpha L_a}{K_c}\right)\right) L_{2_c} \\
L'_{3_c} &= \tau_{L_{2_c}} L_{2_c} - \left(\tau_{L_{3_c}} + \mu_{L_{3_c}} \left(1 + \frac{L_c + \alpha L_a}{K_c}\right)\right) L_{3_c} \\
L'_{4_c} &= \tau_{L_{3_c}} L_{3_c} - \left(\tau_{L_{4_c}} + \mu_{L_{4_c}} \left(1 + \frac{L_c + \alpha L_a}{K_c}\right)\right) L_{4_c} \\
L'_a &= \tau_{E_a} E_a - \left(\tau_{L_a} + \mu_{L_a} \left(1 + \frac{L_a}{K_a}\right)\right) L_a \\
P'_c &= \tau_{L_{4_c}} L_{4_c} - \left(\tau_{P_c} + \mu_{P_c}\right) P_c \\
P'_a &= \tau_{L_a} L_a - \left(\tau_{P_a} + \mu_{P_a}\right) P_a \\
A'_c &= \frac{1}{2} \tau_{P_c} (1-p) P_c - \mu_{A_c} A_c - \chi_C \beta_c A_c \\
A'_a &= \frac{1}{2} \tau_{P_a} P_a - \mu_{A_a} A_c - \chi_C \beta_a A_a \\
C'_c &= \chi_C \beta_c A_c \\
C'_a &= \chi_C \beta_a A_a
\end{aligned}
\qquad (M)
$$

- where $\tau_{E_c}, \tau_{E_a}, \tau_{L_{1_c}}, \tau_{L_{2_c}}, \tau_{L_{3_c}}, \tau_{L_{4_c}}, \tau_{L_a}, \tau_{P_c}, \tau_{P_a}$ are the temperature dependent developmental rates driving the transitions of the two vector species across the different life stages considered;
- $\mu_{E_c}, \mu_{E_a}, \mu_{L_{1_c}}, \mu_{L_{2_c}}, \mu_{L_{3_c}}, \mu_{L_{4_c}}, \mu_{L_a}, \mu_{P_c}, \mu_{P_a}, \mu_{A_c}, \mu_{A_a}$ are the temperature dependent death rates associated with the different stages;
- n_{Ec} and n_{Ea} are the number of eggs laid in one oviposition for a female of *Cx. pipiens* and *Ae. albopictus* respectively;
- d_{Ac} and d_{Aa} are the length of the gonotrophic cycles of the two species;
- K_a and K_c are density-dependent scaling factors driving the carrying capacity for the larval stages;
- p is the probability (depending on daylight duration) that a fully developed *Cx. pipiens* pupa becomes a diapausing adult;
- α represents the increase in mortality of *Cx. pipiens* larval stages due to competition with *Ae. albopictus*. Its value is 0 if the daily temperature is below 15°C.;
- β_a and β_c are the adult capture rates;
- χ_C is a function of time defined equal to 1 when the trap is open and 0 otherwise;
- L_c represents the total *Cx. pipiens* larval population, i.e. $L_c=L_{1c}+L_{2c}+L_{3c}+L_{4c}$;
- C_a and C_c represents the cumulative number of captured female adult mosquitoes for *Ae. albopictus* and *Cx. pipiens* respectively.

Since only female adult mosquitoes are explicitly considered in the model, the term 1/2 in the equation for the adults accounts for the sex ratio (Delatte 2009, Vinogradova 2011). Moreover, given that diapausing *Cx. pipiens* females do not take blood meals before overwintering (Denlinger 2014), they are unlikely to be captured by using the considered traps. Consequently, only non-diapausing female adults are considered in the model.

We implemented model *M* as a discrete–time stochastic version, with time–step $\Delta t = 1$ day, in order to account for the stochastic nature of the processes. Precisely, the model is a Markov chain whose states represent the integer number of individuals in all developmental stages. Transition probabilities are built according to Poisson distributions whose means are obtained from the rate in system *M*. The seasonal dynamics of the mosquito population of each site is simulated from April 1 (corresponding to approximately one month before the first reported capture session) to October 31. Since, to the best of our knowledge, no data are available regarding the overwintering of *Cx. pipiens* and *Ae. albopictus*, we initialize the system with 100 non-diapausing *Cx. pipiens* adults and 10000 *Ae. albopictus* eggs. Preliminary model simulations showed no significant change of the model's behavior for different initial conditions.

Mortality and developmental rates across different vector life stages have been modeled as a function of temperature as published in (Poletti 2011, Marini 2016). The probability p for a developed *Cx. pipiens* pupa to become a diapausing adult is a function of daylight duration as presented in (Marini 2016). The average number of eggs laid n_{Ea} and n_{Ec} per oviposition were fixed to 60 (Poletti 2011) and 190 (Marini 2016) respectively. The duration of the gonotrophic cycle d_{Aa} is a function of temperature as in (Poletti 2011), while d_{Ac} is fixed to 5.54 days (Faraj 2006).

We assumed that, for each capture session, the number of captured female adult mosquitoes follows a Poisson distribution with mean obtained from the model; therefore, for each dataset, the likelihood of the observed data given a parameter set θ has been defined as

$$L = \prod_{i=1}^{h} e^{\{-\tilde{A}_i(\theta)\}} \frac{\tilde{A}_i(\theta)^{A_i}}{A_i!} \cdot e^{\{-\tilde{C}_i(\theta)\}} \frac{\tilde{C}_i(\theta)^{C_i}}{C_i!}$$

where i runs over the number of capture sessions h, A_i (C_i) is the observed number of captured *Ae. albopictus* (*Cx. pipiens*) adults at capture session i and $\widetilde{A}_i(\theta)$ ($\widetilde{C}_i(\theta)$) is the predicted number of captures of *Ae. albopictus* (*Cx. pipiens*) at capture session i simulated by the model with parameters $\theta = (\alpha, \beta, K_a, K_c)$.

Model output

Figure A shows an example of the temporal dynamics of larvae and adults for both species predicted by the two models in a given site and year. During spring months (April and May), the presence of *Ae. albopictus* larvae is limited by the relatively low temperatures and both models predict the same expansion of *Cx. pipiens* larvae and adults. Afterwards (beginning of June), increasing temperatures cause the rise of the *Ae. albopictus* population; consequently, the model with competition predicts a sharp fall of the larval (Fig. Aa) and adult (Fig. Ab) *Cx. pipiens* abundances. On the other hand, with the independent populations model, the decline of *Cx. pipiens* adults begins in late summer (August), when higher temperatures increase their mortality and progressively shortening photoperiods induce diapause in a growing number of newly emerged adult females. *Ae. albopictus* adults are better suited to higher temperatures and do not diapause, therefore their decline does not start until mid-September (Fig. Ab).

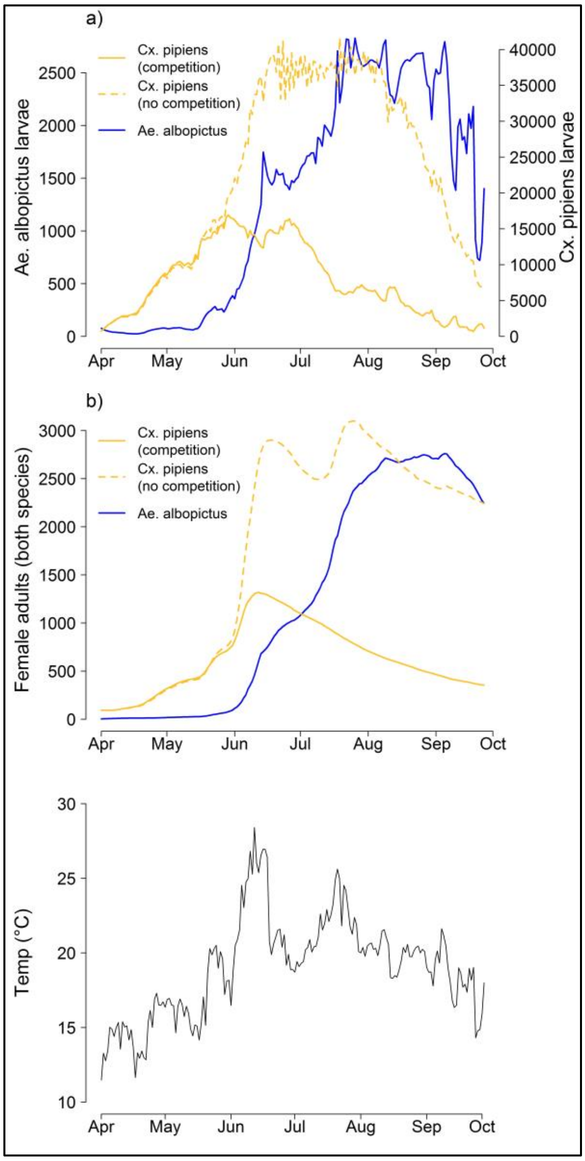

Figure A. **Example of predicted populations**. Example of average larval (a) and adults (b) populations predicted by the two considered models in a selected site. Lower panel shows the recorded temperature.

Model fit

The model fits the observed data quite well. In fact, about 75% of the recorded weekly captures lies in the 95% Credible Interval (CI) of model predictions with both assumptions.

In sites where the competition model was selected, the 95% CI of captured females predicted by the independent population model included the observed *Cx. pipiens* captures in 65% of data points overall, compared to 72% in the competition model. As expected, both models fitted equally well *Ae. albopictus* data, with about 80% of observations lying within the 95% CI of model predictions.

DIC and AIC analysis

We compared the goodness of fit of the model with interspecific competition against that of the model with independent populations (i.e. with α set equal to 0), using the Deviance Information Criterion (DIC):

$$DIC = E(D) + \frac{1}{2}\mathrm{var}(D)$$

where $D = -2\ln L$, *E(D)* is the average value of *D* and *var(D)* is its variance.

Models with smaller DIC should be preferred. In fact, if the likelihood *L* is high (closer to 1) then $\ln L$ is closer to 0. Moreover, $\mathrm{var}(D)$ increases with model complexity: in this way, the DIC penalizes models with a higher number of free parameters. We denote with DIC_α the value obtained with the interspecific competition model and with DIC_0 the value associated with the independent populations model. Generally, model selection using the DIC criterion only requires a model to have a lower DIC than the alternative (corresponding to $\Delta DIC = DIC_0 - DIC_\alpha > 0$) (Spiegelhalter 2002). Considering the high stochastic noise in the capture data and the number of free parameters in our models, we conservatively restricted this criterion in such a way to minimize the risk of false positives on the existence of competition (Spiegelhalter 2002), by fixing a higher threshold on the minimum ΔDIC, i.e. ΔDIC>4. However, since the value of the threshold is arbitrary, we tested the robustness of our results by using a looser threshold, set to ΔDIC>2 as well as a different score function for model selection, namely, the Akaike Information Criterion, AIC (Burnham and Anderson 2002). AIC is defined as

$$AIC = 2K - E(D)$$

Where *K* is the number of the model parameters. Analogously to the DIC criterion, we selected models based on the value of on ΔAIC with respect to three standard threshold values (namely, 2, 4 and 7) (Burnham and Anderson 2002, Burnham 2011).

By loosening the DIC threshold, we included 5 additional datasets in the competition group (see Table A). The AIC yielded results very similar to the DIC, although slightly more conservative for corresponding values of the threshold. As reported in Table A and Figures B and C, qualitative results presented in the main text do not change significantly when applying a different threshold on DIC or when using the AIC with either threshold.

		ΔDIC>2		ΔAIC>2		ΔAIC>4		ΔAIC>7	
	All datasets	Competition (%)	Independent populations (%)	Competition (%)	Independent populations (%)	Competition (%)	Independent populations (%)	Competition (%)	Independent populations (%)
**All years**	73	34 (47%)	39 (53%)	29 (40%)	44 (60%)	23 (32%)	50 (68%)	18 (25%)	55 (75%)
**2014**	39	9 (23%)	30 (77%)	7 (18%)	32 (82%)	5 (13%)	34 (87%)	4 (10%)	35 (90%)
**2015**	34	25 (74%)	9 (26%)	22 (65%)	12 (35%)	18 (53%)	16 (47%)	14 (41%)	20 (59%)

Table A. Number of datasets by year, selected model and DIC and AIC threshold.

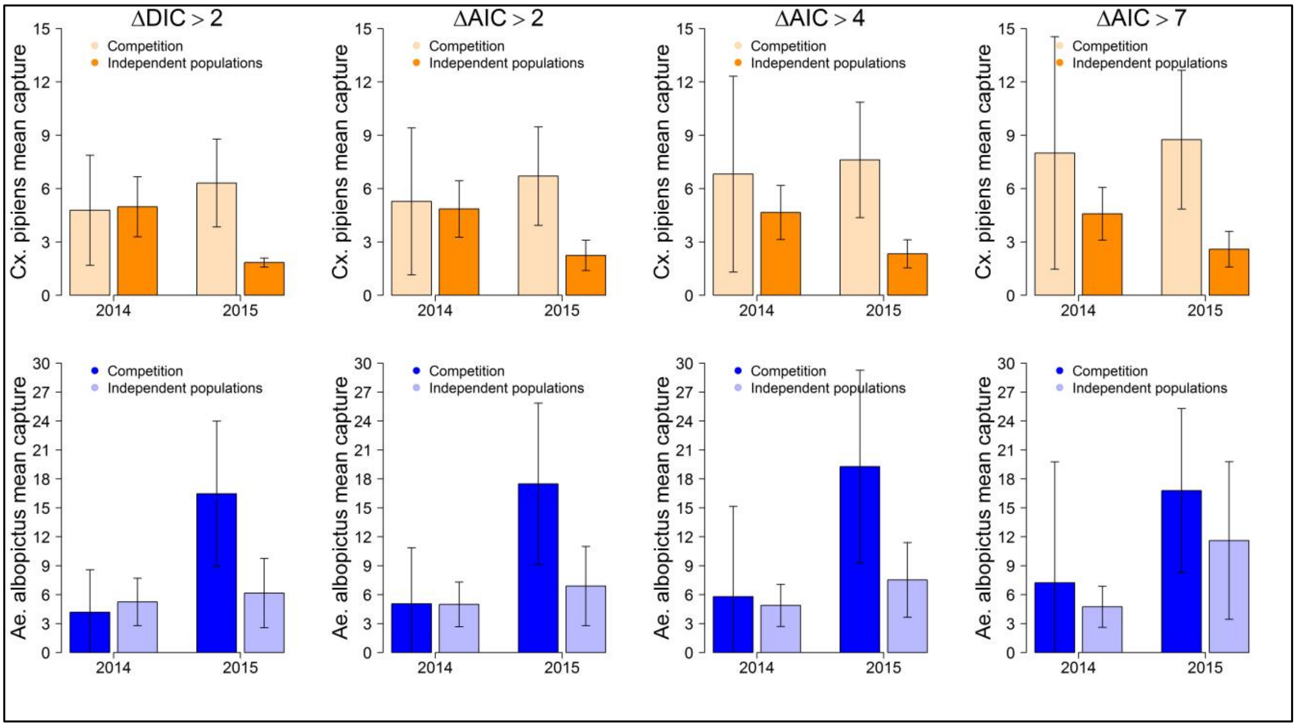

Figure B. Average number of captured *Cx. pipiens* (panels in the upper row) and *Ae. albopictus* (panels in the lower row) per site by criterion of model selection (ΔDIC>2, ΔAIC>2, ΔAIC>4 and ΔAIC>7, from left to right) by selected model and year.

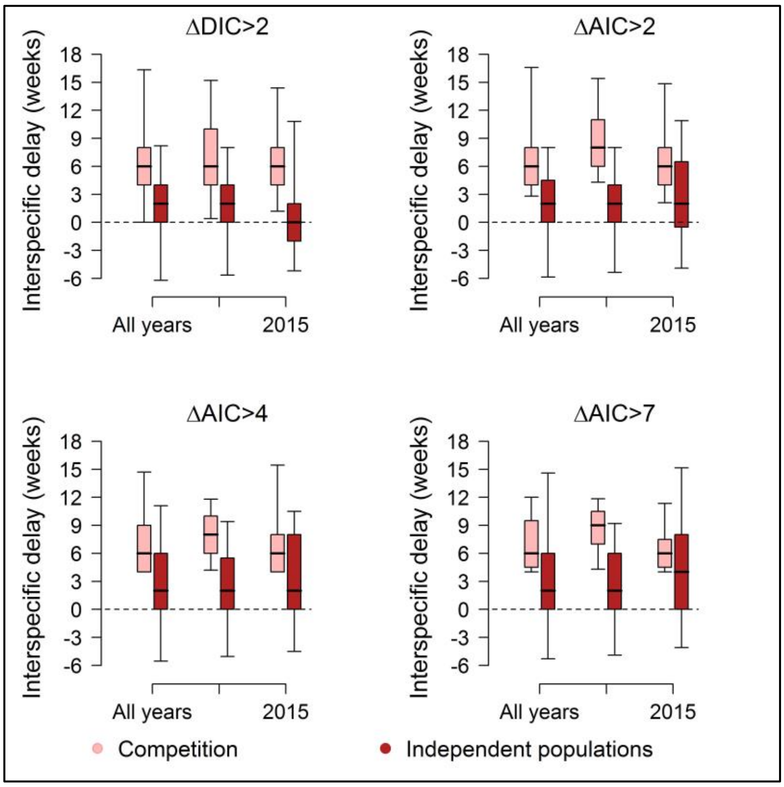

Figure C. The interspecific delay (in weeks, median, quartiles and 95% credible intervals) computed for *Ae. albopictus* and *Cx. pipiens* capture patterns by criterion of model selection. Distributions are shown for all datasets combined and aggregated by year.

Estimates of the competition-dependent additional mortality

The ratio $z = \alpha/K_c$ defines the mortality rate of *Cx. pipiens* larvae due to each additional *Ae. albopictus* larva in the breeding site. Figure D shows that estimates of *z* for sites more strongly associated with competition (ΔDIC>4) are on average significantly higher (t-test p-value<0.05) than those associated to independent populations (ΔDIC<0): in other words, the competition model tended to be rejected when the estimated value of the competition-dependent mortality was closer to zero. The distribution of z values in sites with uncertain attribution (characterized by intermediate values of ΔDIC) was in between the two cases: this result mirrors the fact that competition has a nuanced, rather than an on/off effect on mosquito abundance.

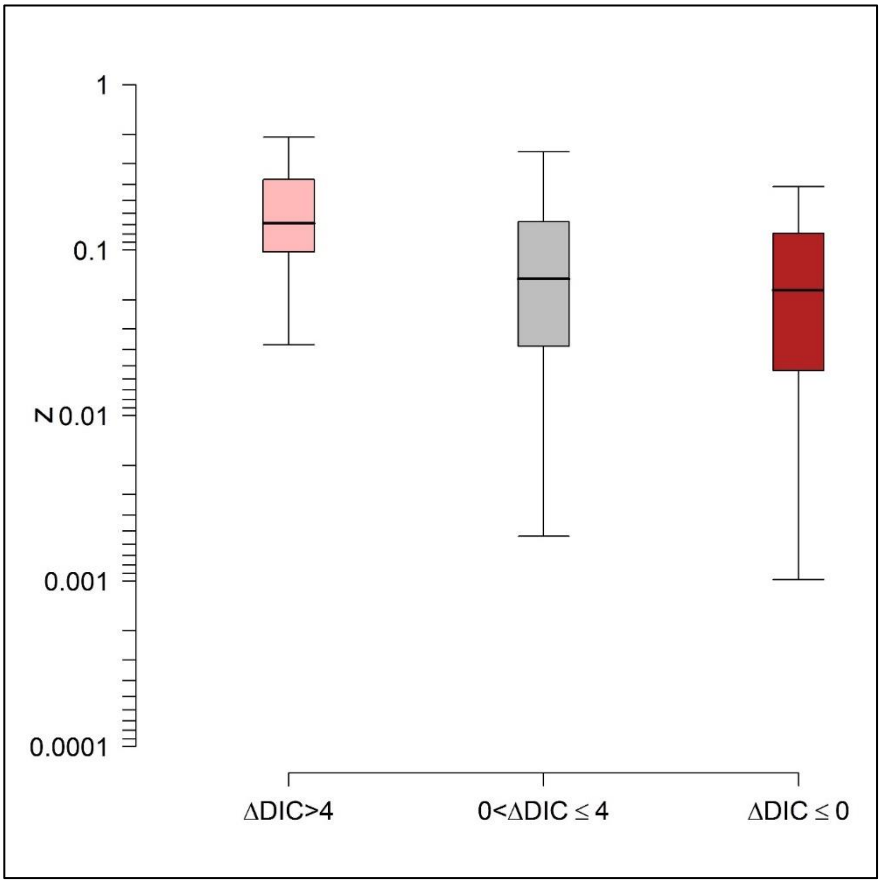

Figure D. Boxplot (2.5%, 25%, 75% and 97.5% quantile and median) of estimated posterior distribution of *z* by ΔDIC.

References

Burnham KP, Anderson DR. Model selection and multimodel inference: a practical information-theoretic approach. New York: Springer; 2002.

Burnham KP, Anderson DR, Huyvaert KP. AIC model selection and multimodel inference in behavioral ecology: some background, observations, and comparisons. Behav Ecol Sociobiol. 2011;65:23–35.

Delatte H, Gimonneau G, Triboire A, Fontenille D. Influence of temperature on immature development, survival, longevity, fecundity, and gonotrophic cycles of Aedes albopictus, vector of chikungunya and dengue in the Indian Ocean. J Med Entomol. 2009; 46(1):33–41.

Denlinger DL, Armbruster PA. Mosquito diapause. Annu Rev Entomol. 2014; 59:73–93.

Marini G, Poletti P, Giacobini M, Pugliese A, Merler S, Rosà R. The Role of Climatic and Density Dependent Factors in Shaping Mosquito Population Dynamics: The Case of Culex pipiens in Northwestern Italy. PLOS ONE. 2016; 11(4):e0154018.

Poletti P, Messeri G, Ajelli M, Vallorani R, Rizzo C, Merler S. Transmission Potential of Chikungunya Virus and Control Measures: The Case of Italy. PLOS ONE. 2011; 6(5):e18860.

Spiegelhalter DJ, Best NG, Carlin BR, van der Linde A. Bayesian measures of model complexity and fit. J R Stat Soc Ser B-Stat Methodol. 2002; 64:583–616.

Vinogradova EB. The sex structure of the larval populations of the urban mosquito Culex pipiens pipiens f. molestus Forskal (Diptera, Culicidae) in St. Petersburg. Entmol Rev. 2011; 91(6):729–34.
